# Parathyroidectomy and Cardiometabolic Risks in Patients With Primary Hyperparathyroidism

**DOI:** 10.1001/jamanetworkopen.2025.44623

**Published:** 2025-11-21

**Authors:** Nir Tsur, Nofar Edri, Tomer Kerman, Yeela Talmor-Barkan, Shir Kushnir, Gideon Bachar

**Affiliations:** 1 Department of Otolaryngology–Head and Neck Surgery, Rabin Medical Center–Beilinson Hospital, Petach Tikva, Israel; 2 Faculty of Medicine, Tel Aviv University, Tel Aviv, Israel; 3 Joyce & Irving Goldman Medical School, Faculty of Health Sciences, Ben-Gurion University of the Negev, Be’er-Sheva, Israel; 4 Department of Cardiology, Rabin Medical Center, Petah Tiqva, Israel; 5 Research Authority, Rabin Medical Center, Beilinson Campus, Petah Tiqva, Israel

## Abstract

**Importance:**

Primary hyperparathyroidism (pHPT) is a common endocrine disorder linked to elevated parathyroid hormone and calcium levels, which may contribute to increased cardiometabolic risk.

**Objective:**

To evaluate the long-term cardiometabolic risks associated with pHPT and to assess whether parathyroidectomy is associated with reduced incidence of these outcomes.

**Design, Setting, and Participants:**

This retrospective cohort study used data from Clalit Health Services, Israel’s largest health care organization. A total of 50 199 patients diagnosed with pHPT between January 1, 2000, and November 29, 2023, and 150 265 matched controls (matched 1:3 by age, sex, and socioeconomic status) were included. Patients with secondary hyperparathyroidism were excluded. Among patients with pHPT, 6654 underwent parathyroidectomy. Duration of follow-up was as long as 15 years. Data were analyzed from November 1, 2024, to April 1, 2025.

**Exposures:**

Diagnosis of pHPT and parathyroidectomy.

**Main Outcomes and Measures:**

Incidence of hypertension, type 2 diabetes (T2D), cardiovascular disease (CVD), and cerebrovascular accidents (CVA). Events were analyzed using Kaplan-Meier curves and multivariable-stratified Cox proportional hazards regression models adjusted for Charlson Comorbidity Index, body mass index, and ethnicity.

**Results:**

A total of 200 464 individuals were included (median age, 66 [IQR, 55-75] years; 136 884 [68.3%] female), comprising 50 199 patients with pHPT and 150 265 matched control individuals. Patients with pHPT exhibited an elevated long-term risk of hypertension (adjusted hazard ratio [AHR], 1.22; 95% CI, 1.17-1.33; *P* < .001), T2D (AHR, 1.07; 95% CI, 1.01-1.16; *P* = .02), CVD (AHR, 1.28; 95% CI, 1.21-1.42; *P* < .001), and CVA (AHR, 1.22; 95% CI, 1.17-1.33; *P* < .001) compared with controls. Between patients who underwent parathyroidectomy and those who underwent nonsurgical management, parathyroidectomy was associated with a reduced risk of T2D only, with the incidence decreasing from 15.18 (95% CI, 14.58-15.81) to 10.77 (95% CI, 9.80-11.83) per 1000 person-years (AHR, 0.56; 95% CI, 0.30-0.89; *P* = .002).

**Conclusions and Relevance:**

The findings of this cohort study suggest that pHPT is associated with increased long-term cardiometabolic risks. Parathyroidectomy may be associated with reduced incidence of T2D, supporting consideration of surgical intervention in appropriate patients.

## Key Points

**Question:**

Is primary hyperparathyroidism (pHPT) associated with increased cardiometabolic risks, and might parathyroidectomy help reduce these risks?

**Findings:**

In this cohort study including 50 199 patients with pHPT and 150 265 matched controls, pHPT was associated with significantly increased incidence of hypertension, type 2 diabetes, cardiovascular disease, and cerebrovascular accident. Among 6654 patients with pHPT who underwent parathyroidectomy, the incidence of type 2 diabetes was significantly lower compared with those receiving nonsurgical treatment; no differences were found for other types of cardiometabolic risk.

**Meaning:**

These findings suggest that parathyroidectomy may be associated with reduced risk of type 2 diabetes in patients with pHPT.

## Teaser

This cohort study assesses the prevalence and severity of cardiovascular complications in patients with primary hyperparathyroidism and examines whether parathyroidectomy is associated with improved cardiometabolic outcomes among these patients.

## Introduction

Primary hyperparathyroidism (pHPT) is the third most common endocrine disorder worldwide, characterized by the overproduction of parathyroid hormone (PTH), which often leads to hypercalcemia and a range of systemic complications.^1,2,3^ Among the most serious complications are the cardiovascular risks associated with pHPT, including hypertension,^4^ arrhythmias, and vascular calcification, which impair patient well-being and quality of life. Although several studies have demonstrated the prevalence of these complications, the extent, underlying mechanisms, and long-term outcomes remain underexplored.^3,5,6,7,8,9^ There is ongoing debate regarding the clinical importance of these cardiovascular outcomes and the necessity of routine cardiovascular assessment in patients with pHPT.^10,11^

Parathyroidectomy effectively normalizes PTH and calcium levels, correcting metabolic abnormalities.^12^ However, its impact on long-term cardiovascular risks remains uncertain.^13,14,15,16^ While early intervention is thought to reduce these risks, its effectiveness varies substantially depending on patient demographics, disease severity, and clinical presentation.^16,17,18^ Some studies suggest cardiovascular risks in patients with pHPT can persist or only partially improve after achieving postsurgical normocalcemia.^18,19,20^ These variations highlight the complexity of pHPT as a systemic disease and underscore the need for a deeper understanding of the implications of excess PTH levels concerning cardiovascular morbidity.

This study aims to assess the prevalence and severity of cardiovascular complications in patients with pHPT. Additionally, we examine the association of parathyroidectomy with these cardiovascular risks and metabolic outcomes.

## Methods

### Study Design and Population

This population-based cohort study was conducted at Rabin Medical Center, a university-affiliated hospital run by Clalit Health Service (CHS), the largest of 4 state-mandated health management organizations in Israel with approximately 4.4 million members. The CHS database includes information on diagnoses, hospitalizations, clinic visits, demographic characteristics of patients, laboratory values, and medications. Members’ low turnover (approximately 1%) enables accurate long-term follow-up. The study was conducted in accordance with the Declaration of Helsinki^21^ and was approved by the Ethics Committee of Rabin Medical Center, which waived the need for informed consent due to the retrospective nature of the study and the use of deidentified data. The study followed the Strengthening the Reporting of Observational Studies in Epidemiology (STROBE) reporting guideline.

The study cohort consisted of patients 18 years or older registered with CHS between January 1, 2000, and November 29, 2023. The study group included patients with pHPT, defined by elevated PTH levels, excluding secondary causes such as chronic kidney disease, vitamin D deficiency, or malabsorption. The control group included individuals without pHPT, matched in a 1:3 ratio by birth year, sex, and socioeconomic status (SES).

### Variables and Data Collection

Data on cardiovascular-related conditions were extracted, specifically the first recorded diagnoses of hypertension, type 2 diabetes (T2D), cardiovascular diseases (CVD; including ischemic heart disease, acute myocardial infarction, and heart failure), and cerebrovascular accidents (CVA; including cerebral infarction). Additional demographic, clinical, and laboratory data, including age, sex, ethnicity, SES (low, medium, or high), smoking status, body mass index (BMI), and laboratory results, were also extracted. The Charlson Comorbidity Index (CCI) was calculated for each patient to assess the burden of comorbid conditions. SES was classified according to the Israeli Central Bureau of Statistics methodology.^22^ Ethnicity was assessed based on national registry data and reported using standard categories (Jewish or Arab); these data were collected to account for potential confounding by ethnicity, as Jewish and Arab populations in Israel represent 2 distinct groups that have been shown to differ in demographic and clinical characteristics.^23^ Sex was reported as male or female (no other categories were recorded). Patients with missing baseline data or lost to follow-up were excluded from each relevant analysis. All data were obtained through the CHS data-sharing platform powered by MDClone.^24^

### Outcome Definitions

Incident cases of hypertension, T2D, CVD (ischemic heart disease, myocardial infarction, and heart failure), and CVA were identified using *International Classification of Disease, Ninth Revision* (*ICD-9*), diagnostic codes registered in the CHS database. For T2D, ascertainment required a new *ICD-9* diagnosis code in patients without a prior T2D diagnosis, without prior use of antidiabetic medication, and with normal hemoglobin A_1c_ levels (<6.5%) prior to the index date. CVD and CVA outcomes were defined based on validated *ICD-9* coding algorithms that were previously applied in CHS research.^25^ Only first incident events were considered, and patients with preexisting diagnoses of the respective outcome were excluded from each outcome-specific analysis.

### Statistical Analysis

Data were analyzed from November 1, 2024, to April 1, 2025. All statistical tests were 2 tailed, and *P* < .05 was considered statistically significant. Data were analyzed using R software, version.3.6.1 (R Program for Statistical Computing).

#### Patients With pHPT vs Matched Controls

Continuous variables were summarized using means and SDs and medians and IQRs, while frequencies and percentages represented categorical variables. Standardized mean differences (SMDs) were calculated for all baseline covariates, with values less than 0.10 indicating good balance between groups.

The incidence rates of cardiovascular-related conditions (hypertension, T2D, CVD, and CVA) were compared between the 2 groups. Each condition was analyzed separately, and individuals with a prior diagnosis of that specific condition were excluded from the analysis of that outcome. This approach ensured that only first incident events were assessed, thereby preventing distortion of the results by individuals at higher baseline risk of recurrence and preserving comparability between groups.

For the study group, follow-up began at the time of pHPT diagnosis, while for the control group, follow-up began at the diagnosis date of the matched case. Follow-up was censored at death, parathyroidectomy, the end of the study period (November 29, 2023), or after 15 years of follow-up. Incident rates per 1000 person-years and 95% CIs were calculated to account for varying follow-up durations. Cumulative incidence was estimated using the 1-minus Kaplan-Meier method, and differences between the survival curves were assessed using the log-rank test. Stratified Cox proportional hazards regression models were applied for each condition with stratification by the matching factors (birth year, sex, and SES) to account for the matched study design. The models were further adjusted for CCI, ethnicity, and BMI. The proportional hazards assumption was evaluated using Schoenfeld residuals and graphical diagnostics, with no major violations observed. Robust CIs were obtained using nonparametric bootstrapping (1000 replications) to ensure stable variance estimation.

#### Parathyroidectomy vs Nonsurgical Management

Patients with pHPT who underwent parathyroidectomy were compared with those who received nonsurgical treatment. Each parathyroidectomy case was matched in a 1:3 ratio to controls by age at diagnosis, sex, and SES. Consistent with the approach used in the patient vs control analysis, each cardiovascular condition was analyzed separately, excluding individuals with a prior diagnosis of the respective condition. Time-dependent stratified Cox proportional hazards regression models were then applied, with parathyroidectomy status included as a time-varying covariate and stratification by the matched sets. The models were further adjusted for CCI, ethnicity, BMI, corrected calcium level, and PTH. Robust CIs were derived using nonparametric bootstrapping with 1000 replications to ensure stable variance estimation.

## Results

### Status at Diagnosis

A total of 200 464 individuals were included in the study (median age, 66 [IQR, 55-75] years; 136 884 [68.3%] female and 63 580 [31.7%] male): 50 199 patients in the pHPT group and 150 265 matched controls. Both groups were well-matched for age, sex, and SES. The CCI showed more pronounced comorbidities in the pHPT group: 32 082 of 50 189 (63.9%) had a comorbidity score of 3 or greater compared with 83 932 of 150 241 (55.9%) in the control group (eFigure in Supplement 1). Notably, being in the pHPT group was associated with higher rates of preexisting hypertension (29 463 [58.7%] vs 67 229 [44.7%]), T2D (9047 [18.0%] vs 17 927 [11.9%]), CVD (11 954 [23.8%] vs 25240 [16.8%]), and CVA (5651 [11.3%] vs 12 321 [8.2%]). These values describe the entire cohort at baseline. In the outcome-specific analyses, patients with a prior diagnosis of the respective condition were excluded. Further baseline characteristics and metabolic values are presented in Table 1.

**Table 1.** Baseline Characteristics of Patients With pHPT and Matched Controls

Characteristic	Control group (n = 150 265)	pHPT group (n = 50 199)	SMD
Age, y			
Mean (SD)	64 (14)	64 (14)	0
Median (IQR)	66 (55-75)	66 (55-75)	
Range	18-104	18-104	
Sex, No./total No. (%)			
Female	102 591/150 265 (68.3)	34 293/50 199 (68.3)	0
Male	47 674/150 265 (31.7)	15 906/50 199 (31.7)	
Ethnicity, No./total No. (%)			
Jewish	124 133/141 180 (87.9)	42 300/46 945 (90.1)	0.07
Arab	17 047/141 180 (12.1)	4645/46 945 (9.9)	
SES, No./total No. (%)			
Low	19 620/143 069 (13.7)	6556/47 791 (13.7)	0
Medium	90 758/143 069 (63.4)	30 279/47 791 (63.4)	
High	32 691/143 069 (22.8)	10 956/47 791 (22.9)	
CCI, No./total No. (%)			
0	18 753/150 241 (12.5)	4621/50 189 (9.2)	0.17
1-2	47 556/150 241 (31.7)	13 486/50 189 (26.9)	
≥ 3	83 932/150 241 (55.9)	32 082/50 189 (63.9)	
Background diagnoses, No./total No. (%)			
Hypertension	67 229/150 265 (44.7)	29 463/46 945 (58.7)	0.28
T2D	17 927/150 265 (11.9)	9047/46 945 (18.0)	0.17
CVD	25 240150 265 (16.8)	11 954/46 945 (23.8)	0.18
CVA	12 321150 265 (8.2)	565146 945 (11.3)	0.10
Smoking, No./total No. (%)	15 899/97 154 (16.4)	4745/32 868 (14.4)	0.05
BMI			
Total No. of participants	109 782	38 337	NA
Mean (SD)	28 (5.7)	28.8 (6.1)	0.13
Median (IQR)	27.3 (24.2-30.9)	27.9 (24.6-31.9)	
Range	10.2-64.9	11.6-65.0	
Calcium level, mg/dL			
Total No. of participants	144 207	49 702	NA
Mean (SD)	9.3 (0.4)	9.8 (1.0)	0.66
Median (IQR)	9.3 (9.0-9.5)	9.7 (9.1-10.4)	
Range	3.0-17.5	3.7-21.2	
Phosphorus level, mg/dL			
Total No. of participants	143 852	49 794	NA
Mean (SD)	3.6 (0.6)	3.4 (0.9)	0.16
Median (IQR)	3.6 (3.2-3.9)	3.3 (2.9-3.8)	
Range	0.8-13.2	0.4-16.8	
PTH level, pg/mL			
Total No. of participants	32 702	46 247	NA
Mean (SD)	57.8 (73.6)	144.5 (189.8)	0.60
Median (IQR)	41.9 (28.7-64.4)	98.0 (64.3-149.0)	
Range	0-2379.0	0-2487.0	
Urine calcium level, mg/24 h			
Total No. of participants	21 524	33 562	NA
Mean (SD)	126.6 (100.6)	172.8 (157.1)	0.35
Median (IQR)	109.0 (56.0-175.5)	136.0 (51.0-256.0)	
Range	0-2473.0	0-2238/6	

Abbreviations: BMI, body mass index (calculated as weight in kilograms divided by height in meters squared; CCI, Charlson Comorbidity Index; CVA, cerebrovascular accident; CVD, cardiovascular disease; NA, not applicable; pHPT, primary hyperparathyroidism; PTH, parathyroid hormone; SES, socioeconomic status; SMD, standardized mean difference; T2D, type 2 diabetes.

SI conversion factors: To convert calcium to mmol/L, multiply by 0.25; phosphorus to mmol/L, multiply by 0.323; PTH to ng/L, multiply by 1.

### Status During Follow-Up

Table 2 provides an overview of cardiovascular-related outcomes in patients with pHPT compared with matched controls. During the follow-up period, incidence rates per 1000 person-years were significantly higher in the pHPT group across all outcomes: hypertension (55.3 [95% CI, 53.97-56.67] vs 44.56 [95% CI, 44.02-45.11]), T2D (16.99 [95% CI, 16.51-17.49] vs 12.81 [95% CI, 12.60-13.03]), CVD (16.09 [95% CI, 15.60-16.59] vs 11.47 [95% CI, 11.26-11.68]), and CVA (19.35 [95% CI, 18.85-19.87] vs 14.10 [95% CI, 13.88-14.32]). Adjusted hazard ratios (AHRs) confirmed the elevated risk in the pHPT group for hypertension (1.22; 95% CI, 1.17-1.33]), T2D (1.07; 95% CI, 1.01-1.16), CVD (1.28; 95% CI, 1.21-1.42), and CVA (1.22; 95% CI, 1.17-1.33). The Figure shows the cumulative incidences of these outcomes, with corresponding tabular data in eTables 1 to 4 in Supplement 1.

**Table 2.** Clinical Outcomes Among Patients With pHPT vs Matched Controls

Characteristic	Control group (n = 150 265)	pHPT group (n = 50 199)	*P* value
Time at risk, y			
Mean (SD)	8.3 (5.4)	6.8 (5.3)	NA
Median (IQR)	8 (3.1-14.5)	5.5 (1.8-11.9)	NA
Range	0-15	0-15	NA
**Hypertension**			
No. of participants	83 036	20 736	NA
No. of events, No. (%)	25 860 (31.1)	6438 (31.0)	NA
Incidence per 1000 person-years (95% CI)	44.56 (44.02-45.11)	55.3 (53.97-56.67)	NA
Unadjusted HR (95% CI)	1 [Reference]	1.36 (1.34-1.47)	<.001
Adjusted HR (95% CI)^a^	1 [Reference]	1.22 (1.17-1.33)	<.001
**T2D **			
No. of participants	132 338	41 152	NA
No. of events, No. (%)	13 657 (10.3)	4661 (11.3)	NA
Incidence per 1000 person-years (95% CI)	12.81 (12.60-13.03)	16.99 (16.51-17.49)	NA
Unadjusted HR (95% CI)	1 [Reference]	1.33 (1.31-1.45)	<.001
Adjusted HR (95% CI)^a^	1 [Reference]	1.07 (1.01-1.16)	.02
**CVD**			
No. of participants	125 025	38 245	NA
No. of events, No. (%)	11 622 (9.3)	4061 (10.6)	NA
Incidence per 1000 person-years (95% CI)	11.47 (11.26-11.68)	16.09 (15.60-16.59)	NA
Unadjusted HR (95% CI)	1 [Reference]	1.43 (1.41-1.57)	<.001
Adjusted HR (95% CI)^a^	1 [Reference]	1.28 (1.21-1.40)	<.001
**CVA**			
No. of participants	137 944	44 548	NA
No. of events, No. (%)	15 670 (11.4)	5625 (12.6)	NA
Incidence per 1000 person-years (95% CI)	14.10 (13.88-14.32)	19.35 (18.85-19.87)	NA
Unadjusted HR (95% CI)	1 [Reference]	1.36 (1.34-1.47)	<.001
Adjusted HR (95% CI)^a^	1 [Reference]	1.22 (1.17-1.33)	<.001

Abbreviations: CVA, cerebrovascular accident; CVD, cardiovascular disease; HR, hazard ratio; NA, not applicable; pHPT, primary hyperparathyroidism; T2D, type 2 diabetes.

^a^ Adjusted for Charlson Comorbidity Index, ethnicity, and body mass index.

**Figure.**
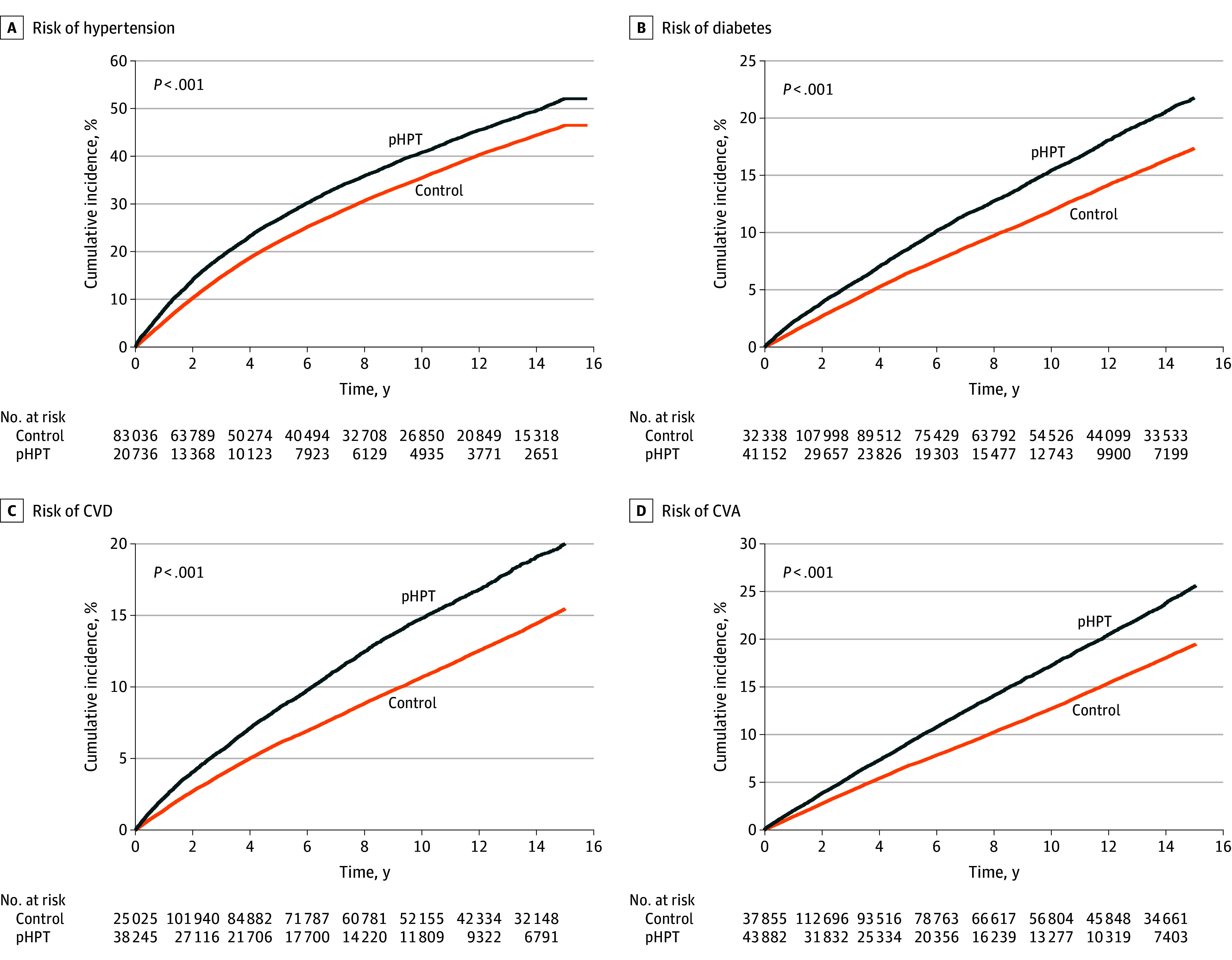
Estimated Cardiometabolic Risk Among Patients With Primary Hyperparathyroidism and Matched Controls Kaplan-Meier survival curves are shown for the development of hypertension (A), type 2 diabetes (B), cardiovascular disease (CVD) (C), and cerebrovascular accident (CVA) (D) in patients with primary hyperparathyroidism (pHPT) and matched controls. Estimates were adjusted for age, sex, body mass index, smoking status, and relevant comorbidities.

### Nonsurgical Treatment vs Parathyroidectomy

The matched cohort included 6654 patients who underwent parathyroidectomy and 19 962 patients who did not undergo surgery. Baseline characteristics were well balanced between groups, with SMDs less than 0.10 for age, sex, ethnicity, and SES. The mean (SD) age was 58 (12) years in both groups. In the nonsurgical management group, 14 403 patients (72.2%) were female and 5559 (27.8%) were male; in the parathyroidectomy group, 4801 (72.2%) were female and 1853 (27.8%) were male. Comorbidity burden and background diagnoses were comparable, with only minor imbalances that remained within acceptable thresholds for matched analyses. Among patients who underwent parathyroidectomy, the mean (SD) time from diagnosis to surgery was 879 (1219) days (Table 3).

**Table 3.** Baseline Characteristics of Patients With Nonsurgical Treatment vs Parathyroidectomy

Characteristic	Nonsurgical treatment (n = 19 962)	Parathyroidectomy (n = 6654)	SMD
Age, y			
Mean (SD)	58 (12)	58 (12)	0
Median (IQR)	59 (51-67)	59 (51-67)	
Range	18-94	19-94	
Sex, No./total No. (%)			
Female	14 403/19 962 (72.2)	4801/6654 (72.2)	0
Male	5559/19 962 (27.8)	1853/6654 (27.8)	
Ethnicity, No./total No. (%)			
Jewish	2105/19 140 (11.0)	626/6371 (9.8)	0.04
Arab	17 035/19 140 (89.0)	5745/6371 (90.2)	
SES, No./total No. (%)			
Low	2331/19 023 (12.3)	731/6341 (11.5)	0.02
Medium	12 506/19 023 (65.7)	4192/6341 (66.1)	
High	4186/19 023 (22.0)	1418/6341 (22.4)	
CCI, No./total No. (%)			
0	2574/19 955 (12.9)	985/6653 (14.8)	0.17
1-2	7524/19 995 (37.7)	2955/6653 (44.4)	
≥ 3	9857/19 995 (49.4)	2713/6653 (40.8)	
Background diagnoses, No./total No. (%)			
Hypertension	9944/19 962 (49.8)	2820/6654 (42.4)	0.15
T2D	2919/19 962 (14.6)	638/6654 (9.6)	0.15
CVD	3385/19 962 (17.0)	826/6654 (12.4)	0.13
CVA	1634/19 962 (8.2)	285/6654 (4.2)	0.15
Smoking, No./total No. (%)	2146/13 785 (15.6)	701/3824 (18.3)	0.07
BMI			
Total No. of participants	15 329	4173	NA
Mean (SD)	29.0 (6.4)	28.4 (5.7)	0.11
Median (IQR)	28.0 (24.5-32.4)	27.6 (24.6-31.5)	
Range	13.3-65.0	14.9-64.9	
Calcium level, mg/dL			
Total No. of participants	19 784	6596	NA
Mean (SD)	9.62 (0.93)	10.52 (1.00)	0.93
Median (IQR)	9.53 (9.05-10.26)	10.54 (10.04-11.02)	
Range	3.74-19.41	3.74-21.16	
Phosphorus level, mg/dL			
Total No. of participants	19 794	6617	NA
Mean (SD)	3.53 (0.93)	3.05 (0.86)	0.54
Median (IQR)	3.40 (2.97-3.90)	2.90 (2.53-3.32)	
Range	0.71-14.29	0.90-12.89	
PTH level, pg/mL			
Total No. of participants	18 469	6241	NA
Mean (SD)	136 (181)	155 (211)	0.10
Median (IQR)	94 (62-137)	106 (65-168)	
Range	0-2464	0-2487	
Urine calcium level, mg/24 h			
Total No. of participants	13 236	5789	NA
Mean (SD)	170 (154)	266 (183)	0.57
Median (IQR)	137 (53-248)	252 (128-378)	
Range	0-2239	0-2141	

Abbreviations: BMI, body mass index (calculated as weight in kilograms divided by height in meters squared); CCI, Charlson Comorbidity Index; CVA, cardiovascular accident; CVD, cardiovascular disease; NA, not applicable; PTH, parathyroid hormone; SES, socioeconomic status; SMD, standardized mean difference; T2D, type 2 diabetes.

SI conversion factors: To convert calcium to mmol/L, multiply by 0.25; phosphorus to mmol/L, multiply by 0.323; PTH to ng/L, multiply by 1.

### Outcomes After Parathyroidectomy

Table 4 provides a detailed comparison of outcomes in patients with pHPT who underwent parathyroidectomy vs those who underwent nonsurgical management. During follow-up, T2D incidence was lower in the surgical group (10.77 [95% CI, 9.80-11.83] vs 15.18 [95% CI, 14.58-15.81] per 1000 person-years; AHR, 0.56 [95% CI, 0.30-0.89]; *P* = .002). For CVA, incidence was 12.93 (95% CI, 11.92-14.03) vs 14.12 (95% CI, 13.55-14.70) per 1000 person-years (AHR, 0.79; 95% CI, 0.50-1.13; *P* = .09), indicating a nonsignificant pattern. CVD occurred at an incidence of 11.57 (95% CI, 10.57-12.66) vs 12.49 (95% CI, 11.94-13.07) per 1000 person-years (AHR, 0.93; 95% CI, 0.85-1.02; *P* = .92), and hypertension at an incidence of 45.89 (95% CI, 42.99-49.00) vs 48.85 (95% CI, 47.32-50.43) per 1000 person-years (AHR, 1.19; 95% CI, 0.69-1.96; *P* = .33), with no significant differences between groups.

**Table 4.** Clinical Outcomes for Patients With Nonsurgical Treatment vs Parathyroidectomy

Characteristic	Nonsurgical treatment	Parathyroidectomy	*P* value
Time at risk, y			
Mean (SD)	6.9 (5.6)	9.1 (5.1)	NA
Median (IQR)	5.7 (1.5-12.8)	9.8 (4.4-15)	NA
Range	0-15	0-15	NA
**Hypertension**			
Incidence per 1000 person-years (95% CI)	48.85 (47.32-50.43)	45.89 (42.99-49.00)	NA
Unadjusted HR (95% CI)	1 [Reference]	1.12 (0.94-1.35)	.07
Adjusted HR (95% CI)^a^	1 [Reference]	1.19 (0.69-1.96)	.33
**T2D**			
Incidence per 1000 person-years (95% CI)	15.18 (14.58-15.81)	10.77 (9.8011.83)	NA
Unadjusted HR (95% CI)	1 [Reference]	0.80 (0.65-0.93)	.001
Adjusted HR (95% CI)^a^	1 [Reference]	0.56 (0.30-0.89)	.002
**CVD**			
Incidence per 1000 person-years (95% CI)	12.49 (11.94-13.07)	11.57 (10.57-12.66)	NA
Unadjusted HR (95% CI)	1 [Reference]	0.95 (0.78-1.13)	.42
Adjusted HR (95% CI)^a^	1 [Reference]	0.93 (0.85-1.02)	.92
**CVA**			
Incidence per 1000 person-years (95% CI)	14.12 (13.55-14.70)	12.93 (11.92-14.03)	NA
Unadjusted HR (95% CI)	1 [Reference]	0.81 (0.67-0.91)	<.001
Adjusted HR (95% CI)^a^	1 [Reference]	0.79 (0.50-1.13)	.09

Abbreviations: CVA, cerebrovascular accident; CVD, cardiovascular disease; HR, hazard ratio; NA, not applicable; T2D, type 2 diabetes.

^a^ Adjusted for Charlson Comorbidity Index, ethnicity, body mass index, corrected calcium level, and parathyroid hormone level.

## Discussion

This nationwide cohort study provides comprehensive insights into the cardiometabolic implications of pHPT and the impact of parathyroidectomy. In our analysis of 50 199 patients with pHPT compared with 150 265 matched controls, we found that pHPT was associated with significantly increased long-term risks of hypertension, T2D, CVD, and CVA. Among the 6654 patients who underwent parathyroidectomy, surgery was associated with a significantly reduced incidence of T2D, whereas no associations were observed for hypertension, CVD, or CVA. These findings highlight both the systemic burden of pHPT and the selective metabolic benefit of surgical intervention.

The observed associations suggest that pHPT contributes to the development and progression of multiple cardiometabolic conditions through systemic effects that extend beyond calcium and bone metabolism. Prolonged elevations in PTH levels have been linked to vascular endothelial dysfunction, insulin resistance, left ventricular hypertrophy, and increased arterial stiffness. These mechanisms may underlie the increased incidence of hypertension, T2D, and atherosclerotic events observed in this cohort, underscoring the importance of early recognition and comprehensive management of pHPT.

Our findings align with previous findings from the literature concerning the cardiovascular burden in patients with pHPT.^4,17^ Antignani et al^4^ highlighted that pHPT contributes to vascular stiffness and hypertension due to hypercalcemia affecting vascular smooth muscle function, an observation that aligns with our reported outcomes. Moreover, Agarwal et al^17^ discussed broader cardiovascular mechanisms, such as vascular calcification and endothelial dysfunction, supporting our observations of the systemic nature of pHPT’s association with cardiovascular health. While these mechanisms are well established, our study quantifies the clinical impact across a broad range of cardiovascular outcomes, particularly T2D and a possible pattern of cerebrovascular events, which have been less emphasized in previous literature. Notably, the large scale of our cohort allows for high-quality comparisons with strong statistical power, positioning it among the most comprehensive retrospective analyses of pHPT-related cardiovascular risk to date.^22^

Besides confirming elevated cardiovascular risk, we provide critical insights into the impact of parathyroidectomy. Our results show that parathyroidectomy was associated with reduced incidence of T2D (AHR, 0.56; 95% CI, 0.30-0.89; *P* = .002). Our results align with those recently reported by Liu et al,^26^ who demonstrated that parathyroidectomy was associated with a lower risk of incident T2D in patients with pHPT, particularly among younger individuals and those with more severe biochemical disease. While their study focused primarily on glycemic outcomes in a smaller cohort, our nationwide analysis extends these observations by evaluating the impact of parathyroidectomy on a broader range of cardiometabolic outcomes in more than 26 000 patients with pHPT. Consistent with the findings reported by Liu et al,^26^ we found that parathyroidectomy was associated with reduced incidence of T2D. However, in contrast to some previous reports,^22,27,28^ no clear benefits were observed for hypertension, CVD, or CVA. This discrepancy may stem from long-term vascular remodeling caused by chronic hypercalcemia and elevated PTH levels, which might not fully reverse after surgery, especially given the prolonged time to intervention (mean 879 [1219] days). Taken together, these findings reinforce the metabolic benefit of surgery on glycemic outcomes while suggesting that other cardiometabolic risks may persist despite surgical intervention.

From a clinical management perspective, whether cardiovascular screening should become routine in pHPT remains unanswered. Despite accumulating evidence of heightened risk, current guidelines do not consistently recommend cardiovascular evaluation in these patients. Our findings highlight the increased cardiovascular risk in patients with pHPT and the potential for parathyroidectomy to mitigate some of these risks, particularly T2D and cerebrovascular events. While these associations suggest possible benefits of integrating cardiovascular risk assessment into the management of pHPT, further prospective studies are needed to determine whether such strategies improve long-term outcomes and to clarify the role of surgical referral practices and health care access in shaping these results. This study was conducted within CHS, the largest integrated health care organization in Israel, which provides universal coverage to more than half of the national population and includes a diverse patient population across ethnic and socioeconomic backgrounds; this fact enhances the potential applicability of our findings to other universal health care systems that care for heterogeneous populations. While all patients in our cohort were covered under the same nationwide health maintenance organization, which reduces heterogeneity in access and insurance coverage, unmeasured factors such as geographic variation, physician referral practices, and patient preferences may still influence surgical decision-making. These aspects could not be fully assessed in our study and warrant further investigation.

### Limitations

The retrospective design of this study introduces several inherent limitations, including potential residual confounding and dependence on the accuracy of medical record data. Although we used a large and comprehensive database, some variables, particularly lifestyle factors (eg, diet, physical activity, smoking), were unavailable, limiting our ability to fully adjust for all relevant confounders. Another limitation is the absence of detailed symptom documentation, which prevented assessment of symptom resolution. To address baseline imbalances, we performed propensity score matching on age, sex, and SES, which achieved good balance across groups (all SMDs <0.10). Nonetheless, residual confounding may persist, particularly due to unmeasured factors such as comorbidity burden, biochemical disease severity, or physician referral practices that may have influenced surgical referral. In addition, excluding patients with preexisting cardiometabolic conditions may have underestimated the overall burden of pHPT, as undiagnosed cases might have been missed. Finally, lack of consistent postoperative calcium and PTH data precluded analysis of whether biochemical normalization mediates risk reduction. These limitations may affect the precision of our estimates.

## Conclusions

In this cohort study of patients with pHPT, the condition was associated with increased long-term cardiometabolic risks. Parathyroidectomy was associated with a significantly reduced incidence of T2D, while no associations were observed for hypertension, CVD, or CVA. These findings support a potential role for surgical intervention in modifying selected cardiometabolic outcomes in this population. Further prospective studies are needed to confirm these observations and to clarify the broader impact of parathyroidectomy on long-term cardiovascular health.

## Notes

Supplement 1. **eTable 1.** Cumulative Incidence of Hypertension in Patients With pHPT and Matched Controls **eTable 2.** Cumulative Incidence of Diabetes in Patients With pHPT and Matched Controls **eTable 3.** Cumulative Incidence of Cardiovascular Disease in Patients With pHPT and Matched Controls **eTable 4.** Cumulative Incidence of Cerebrovascular Accident in Patients With pHPT and Matched Controls **eFigure.** Flowchart of Study Population

Supplement 2.
Data Sharing Statement
