# Supplementary material for: Parathyroidectomy and Cardiometabolic Risks in Patients With Primary Hyperparathyroidism
Source: JAMA Netw Open. 2025 Nov 21;8(11):e2544623. doi: 10.1001/jamanetworkopen.2025.44623 (PMC12639482; doi:10.1001/jamanetworkopen.2025.44623)
Supplement: Supplement 1. — eTable 1. Cumulative Incidence of Hypertension in Patients With pHPT and Matched Controls eTable 2. Cumulative Incidence of Diabetes in Patients With pHPT and Matched Controls eTable 3. Cumulative Incidence of Cardiovascular Disease in Patients With pHPT and Matched Controls eTable 4. Cumulative Incidence of Cerebrovascular Accident in Patients With pHPT and Matched Controls eFigure. Flowchart of Study Population [file jamanetwopen-e2544623-s001.pdf]

Supplementary Online Content

Tsur N, Edri N, Kerman T, Talmor-Barkan Y, Kushner S, Bachar G. Parathyroidectomy and cardiometabolic risks in patients with primary hyperparathyroidism. *JAMA Netw Open*. 2025;8(11):e2543571. doi:10.1001/jamanetworkopen.2025.43571

eTable 1. Cumulative Incidence of Hypertension in Patients With pHPT and Matched Controls
eTable 2. Cumulative Incidence of Diabetes in Patients With pHPT and Matched Controls
eTable 3. Cumulative Incidence of Cardiovascular Disease in Patients With pHPT and Matched Controls
eTable 4. Cumulative Incidence of Cerebrovascular Accident in Patients With pHPT and Matched Controls
eFigure 1. Flowchart of Study Population

This supplementary material has ben provided by the authors to give readers additional information about their work.

© 2025 Tsur N et al. *JAMA Network Open*.

eTable 1. Cumulative Incidence of Hypertension in Patients With pHPT and Matched Controls

Characteristic	1 year	5 years	10 years	15 years	P value
Group, % (95% CI)					<0.001
Control	5.3 (5.1-5.4)	22.1 (21.8-22.4)	35.5 (35.1-35.9)	46.5 (46, 46.9)	
pHPT	7.9 (7.5-8.2)	26.8 (26.1-27.5)	40.8 (39.9-41.6)	52.1 (51-53.1)	

eTable 2. Cumulative Incidence of Diabetes in Patients With pHPT and Matched Controls

Characteristic	1 year	5 years	10 years	15 years	P value
Group, % (95% CI)					<0.001
Control	1.4 (1.3-1.4)	6.5 (6.3-6.6)	11.9 (11.7-12.1)	17.3 (17.1-17.6)	
pHPT	2.2 (2.1-2.4)	8.5 (8.2-8.9)	15.4 (14.9-15.9)	21.7 (21.1-22.4)	

eTable 3. Cumulative Incidence of Cardivascular Disease in Patients With pHPT and Matched Controls

Characteristic	1 year	5 years	10 years	15 years	P value
Group, % (95% CI)					<0.001
Control	1.4 (1.3-1.4)	6 (5.9-6.2)	10.7 (10.5-10.9)	15.4 (15.2-15.7)	
pHPT	2.3 (2.1-2.4)	8.5 (8.2-8.8)	14.8 (14.3-15.3)	20 (19.4-20.6)	

eTable 4. Cumulative Incidence of Cerebrovascular Accident in Patients With pHPT and Matched Controls

Characteristic	1 year	5 years	10 years	15 years	P value
Group, % (95% CI)					<0.001
Control	1.4 (1.3-1.5)	6.7 (6.6-6.9)	12.7 (12.5-13)	19.5 (19.2-19.8)	
pHPT	2 (1.9-2.1)	9.1 (8.8-9.4)	17.3 (16.8-17.7)	25.6 (24.9-26.2)	

eTables 1–4. Cumulative incidence of hypertension (S1), diabetes mellitus (S2), cardiovascular disease (S3), and cerebrovascular accident (S4) in patients with primary hyperparathyroidism (pHPT) compared with matched controls at 1, 5, 10, and 15 years of follow-up. Values are shown as percentages with 95% CIs.

© 2025 Tsur N et al. *JAMA Network Open*.

eFigure 1. Flowchart of Study Population

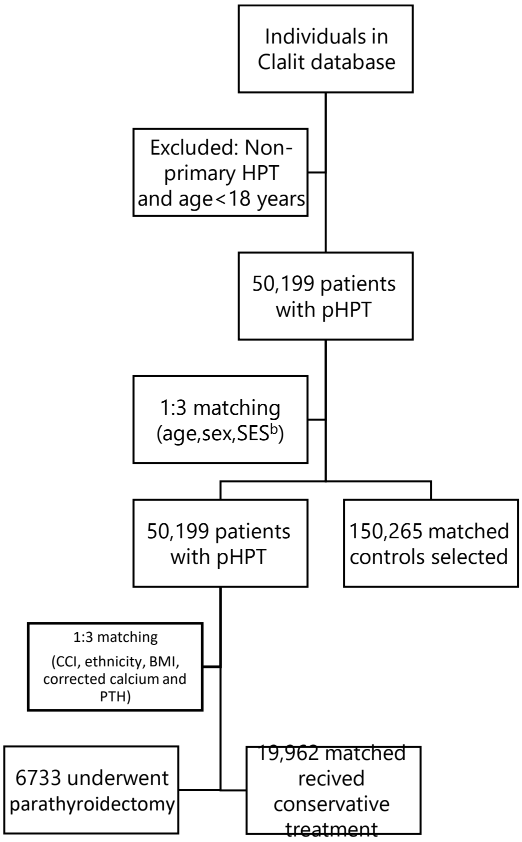

Footnote:

This flowchart illustrates the selection and matching process of patients with primary hyperparathyroidism (pHPT). From an initial population, patients with non-primary HPT and individuals under 18 years of age were excluded, resulting in 50,199 patients with pHPT included for analysis. A 1:3 matching by age, sex, and socioeconomic status (SES) was performed, yielding 50,199 patients diagnosed with pHPT and 150,265 matched controls. Among patients with pHPT, 6,654 underwent parathyroidectomy.

© 2025 Tsur N et al. *JAMA Network Open*.
