# Supplementary material for: Parathyroidectomy and Cardiometabolic Risks in Patients With Primary Hyperparathyroidism
Source: JAMA Netw Open. 2025 Nov 21;8(11):e2544623. doi: 10.1001/jamanetworkopen.2025.44623 (PMC12639482; doi:10.1001/jamanetworkopen.2025.44623)
Supplement: Supplement 2. — Data Sharing Statement [file jamanetwopen-e2544623-s002.pdf]

Data Sharing Statement

Tsur. Parathyroidectomy and Cardiometabolic Risks in Patients With Primary Hyperparathyroidism. *JAMA Netw Open*. Published November 21, 2025.
doi:10.1001/jamanetworkopen.2025.44623

Data
Data available: No
